# Penetrating Wounds of the Chest and Abdomen with Report of a Recent Case

**Published:** 1910-05

**Authors:** C. C. Abell

**Affiliations:** Texarkana, Tex.


### PENETRATING WOUNDS OF THE CHEST AND ABDOMEN WITH REPORT OF A RECENT CASE.

C. C. ABELL, M. D., TEXARKANA, TEX.

Since modern aseptic surgery has made intra-abdominal operations possible, there has been greater progress made in surgery of the abdomen than in any other cavity of the body.

Surgery of the thoracic cavity has improved but little in the last few decades. Estlander's operation is done to-day just as it was when first designed.

Operations upon the cranial cavity date back to the dim distant stone age, when men were trephined or trepanned, as it was then called, with a flint for the purpose of letting out evil spirits and bad humors.

It has only been a few years since the abdomen was considered a sacred cavity, and should not, under any circumstances be invaded.

In surgery of the chest the field is not so wide and fertile as in the abdominal cavity.

Wounds of the heart and great vessels of the chest will proba-probably always be beyond the reach of surgical skill. In wounds of the heart that are not instantly fatal, the pericardinum fills with blood, and the heart fails from compression without immediate surgical intervention. In wounds of the larger vessels that do not kill instantly, the bronchial tubes fill up, the pleural cavity becomes distended, causing death from mechanical interference with respiration. The intercostal artery may be cut down upon and ligated; the internal mammary being a branch of the subclavian, bleeds profusely and being deeper situated, is reached with more difficulty. We are rarely called upon to ligate the pulmonary vessels.

*Signs and Symptoms of Penetrating Wounds of the Chest*.—Bleeding from mouth, cough, bloody expectorations and dyspnœa and by the passage of air in and out through the wound with each respiratory act. There may be pleuritic friction sounds or moist rales. In hemi-thorax there is dullness on percussion, diminished or absent vocal resonance and vocal fremitious. Dyspnoea is due to blood in the bronchial tubes and trachea, and often to partial callapse of the lungs, which is caused by the entrance of air through the walls of the thorax.

*Treatment*.—In the vast majority of cases treatment of penetrating wounds of the chest is simple. Fix and limit the motion of the wounded side with adhesive strips. The circular bandage is desirable in some cases; in others a plaster of Paris dressing, extending from the lower border of the ribs over the shoulders. This confines the breathing principally to the diaphragm, prevents expansion of the lungs, limits or controls bleeding and puts the inflamed parts at rest.

We are indebted to American surgery for the great advances made in the treatment of penetrating wounds of the abdomen. In 1847 Dr. Newall, of New Jersey, did a laparotomy for gun-shot wound of the abdomen suturing the intestine and patient recovered. This operation, at this time, seems to be in a class by itself, for we hear nothing more from it until 1882-84, the same operations with like results were performed in South Carolina. The operation was not brought prominently before the profession until the celebrated paper of Dr. Marion Sims was read before the New York Academy of Medicine in 1881.

In 1884 Dr. Bacon Saunders, of Texas, opened the abdomen for stabbed wound, with symptoms of intra-abdominal hemorrhage, tied the mesenteric artery, which had been divided, with successful result.

A successful result of gun-shot wound of the bladder, the first on record, is to the credit of Dr. A. B. Walker, of Texas. In 1885 Dr. Bull, of New York, operated successfully for multiple perforations of the intestines from a pistol ball.

In 1886 Dr. Roberts, of Louisville, operated successfully for stabbed wound of the intestine. We see that the great state of Texas has contributed her share to the pioneer work in this line.

In the last decade Texarkana has to her credit many penetrating and perforating wounds of the abdomen, successfully treated.

*Diagnosis.*—No distinction should be made between penetrating and perforating wounds of the abdomen. If it is known that penetration has occurred, it is fair to assume that perforation of some of the abdominal viscera has taken place. In incised wounds of the abdomen the diagnosis may be made by inspection, but in stabbed or gun-shot wounds this cannot be done. If gas or intestinal contents escape from the wound, it is clear that the alimentary tract has been perforated. If the wound be in the stomach or duodenum, the gas will have no odor, at least no fecal odor. In this way we can differentiate the wounds of the stomach or duodenum from those of the small or large intestines; by the odorless gas in the one case and the stinking gas in the other. The presence of bile would indicate an injury of the liver, gall bladder, bile duct, possibly the duodenum. Urine would point to the pelvis or the kidney, the ureter or urinary bladder

*Shock and Hemorrhage.*—Hard to differentiate, are often associated together in the same case. In shock we may have immediate collapse; the features are drawn, pale and pinched. The mucous membranes are blanched; the expression dull and apathetic; cerebration is slow and imperfect; the pupils of the eyes dilated and respond but feebly to light; the extremities are cold, there may be nausea and vomiting; the temperature subnormal; pulse may be rapid or slow, irregular and compressible; respiration shallow, irregular and sighing.

*Hemorrhage*—Increased pallor of skin and mucous membranes and increased pulse rate. The pulse becomes more and more feeble and compressible; coldness of the extremities, sometimes cold and clammy perspiration, restlessness, thirst, anxiety, anxious expression, syncope, sometimes vomiting. There may be severe abdominal pains. Rigidity of the muscles may be local or general, rarely distention.

When blood collects in the region of the spleen or below the right lobe of the liver, in the pelvis, in the lesser sac or behind the periteneum and becomes coagulated, or closed in by adhesions, a more or less well-defined palpable mass may be discovered with dullness on percussion in this region. Blood in the right or left illiac fossa will cause dullness on percussion.

We may have a wound from one bullet penetrating both thoracic and abdominal cavities. Such a wound may pierce the pleura, the lungs, the diaphragm, the abdominal cavity and its enclosed organs. Blood and air may be found issuing from the chest wound on respiration. Air from the wounded lung may escape through the diaphragh into the abdominal cavity, causing abdominal distention and loss of liver dullness. Intestinal contents may be drawn up into the thoracic cavity, causing septic pleuritis and death notwithstanding the puncture in the intestines may have been closed and peritonitis prevented. Blood poured into the chest cavity will give the signs of hemo-thorax. A constant bleeding would cause the line of dullness on percussiou to rise higher and higher, as the chest cavity fills with blood. This fluid should not be drawn off at the time, unless it interferes with respiration. Drainage through the ribs may be done, or Estlander's operation may be performed later on, if symptoms of sepsis or empyema develop.

Wounds of the intestines should be closed by sutures or resection, according to whether there is a small perforation or extensive injury. There are thirty or more feet of hollow viscera in the abdomen, and a bullet passing through the cavity below the naval is almost sure to produce from one to twenty or more perforations of their hollow tube.

Stabbed wounds and punctured wounds may produce but a single puncture. In penetrating wounds on a level with, or above the umbilicus, it is possible to have no perforation, especially, if these organs are empty. In the different wars, especially in that between .the states, when soldiers went into battle with empty stomachs, this was occasionally observed.

In looking for wounds of the small intestines, which are more likely to be injured when the abdomen is punctured, it is best to begin at some fixed point, as the duodeno-jejunal junction going from this point to the next fixed point, the ileo-cecal valve, examine for contusions, perforations, injury of the mesentery and mesenteric vessels.

A contused wound of the serous coat caused by a glancing bullet, or a bullet that grazes the intestines, will be followed by a sloughing of the other coats, perforation and death if left alone, hence, these injuries should be treated as perforations. A small wound of the mesentery may be closed with a single suture, but a large wound of the mesentery parallel with the intestine calls for resection. Or a wound of a large mesenteric vessel will be followed by death of that portion of the intestine supplied by the wounded vessel, if left untreated; hence that portion of the intestine should be resected and an end to end anastomosis performed. If more than one wound calls for resection, that portion of the intervening gut between the wounds should be resected, if said wounds are not farther than two or three feet apart. Experience has abundantly shown that one resection is safer than two, even at the sacrifice of a greater amount of intestine.

Dr. Senn has reported four consecutive gun-shot wounds of the abdomen, operated on in the first division hospital corps in Cuba, with four deaths. The brilliant results achieved in private practice have not as yet been paralleled by military surgery, though we are assured that these good results will be forthcoming, but so far, a history of gun-shot wounds of the abdomen, operated upon by military surgeons has given a higher mortality than those left to an expectant plan of treatment. Military surgeons are specialists in gun-shot wounds of the abdomen, as in other portions of the body. Men whose training and education have especially fitted them for work of this nature. Why is it that their results fall so far short of ours? It is their inability to employ a perfectly aseptic technique. It is only in modern hospitals that this can be done to any degree of perfection. In the haste and confusion of battle, and of handling the wounded after battle, without the advantage of a well-equipped hospital, it is found impossible to make this operation a life-saving procedure. By the time they get to a place where any degree of aseptic can be employed, it is too late.

Statistics show that cases operated upon after six or eight hours are attended with a high mortality, rarely recover. The indications are to operate promptly, aseptically, and properly, otherwise, it is better to leave the case to the tender mercies of nature. Even at the risk of delay, the preparation of the patient should be as thorough as the most elaborate abdominal operation of election. We are told by some surgeons, that in cases of alarming intra-abdominal hemorrhage, we might be justified in operating at times, without thorough preparation, but in my mind, this is never justifiable unless immediate death is enevitable without operation. Statistics show that cases operated upon without aseptic details almost always die.

Given aseptic control, surgical skill, and a sufficient number of trained assistants, operate promptly, aseptically and properly.

*Report of Case.*—Was called to see Mr. P., age 44, at 1:30 A. M., May 12th, 1909. I found him over a vessel as if he were at stool, the blood was passing from his bowels in large quantities. He was lifted on the bed, and a hasty examination showed that the bullet had penetrated the right chest wall at the fifth or sixth interspace, and passed down into the abdominal cavity. He was suffering severely from shock and loss of blood. We hurried him to my private sanitarium where a hasty preparation for abdominal operation was made.

On opening the abdomen the blood under tension spurted up with great force. The cavity was filled with blood and hemorrhage still active. Bleeding points were sought out and ligated, and the search for perforations was begun. It was found that the bullet which had entered the right side of the chest, had perforated the pleur and lung, cutting blood vessels and causing hemo-thorax. The bullet passed out of the thorax through the diaphragm into the right hypochon driac region, wounding the liver, stomach, duodenum, producing two perforations in the transverse colon, and many in the small intestines, and lodging in the outer aspect of the left leg about the junction of the middle and upper third.

At the time he received the wound, the stomach and intestines were full, he having eaten a hearty mid-night lunch, and the extravasation was very great. The stomach had crawled over as far as possible into the left hypochondriac region, illustrating the fact that the wounded alimentary tract, like a wounded snake, will writhe and twist and crawl all over the abdominal cavity. Owing to this fact, an intestine which is normally on one side of the abdomen may be found on the opposite side.

The ball, a 38 or 41 calibre, travelled diagonally through Mr. P.'s abdomen, entering at the right upper quadrant, and making its exit at the left lower quadrant. All the bleeding parts were caught up and ligated; wounds of the viscera treated in the usual way. The incision, a long one, extending from the ensiform cartilage to the symphysis pubes in order to reach all fixed and movable parts injured. The extravasation and large quantities of drainage necessary in a wound of this nature, were naturally followed by more or less hernia, but the patient is alive and well to-day. We can readily see what a little delay would have meant to this patient.

